# Palatable Noodles as a Functional Staple Food Made Exclusively from Yellow Peas Suppressed Rapid Postprandial Glucose Increase

**DOI:** 10.3390/nu12061839

**Published:** 2020-06-19

**Authors:** Joto Yoshimoto, Yukiko Kato, Masayasu Ban, Mikiya Kishi, Humitoshi Horie, Chizumi Yamada, Yasuhiro Nishizaki

**Affiliations:** 1 Central Research Institute, Mizkan Holdings Co., Ltd. 2-6 Nakamura-Cho, Handa-Shi, Aichi 475-8585, Japan; yukiko_kato@mizkan.co.jp (Y.K.); mban@mizkan.co.jp (M.B.); mkishi1@mizkan.co.jp (M.K.); 2 Public Interest Incorporated Foundation Aiseikai Aisei Hospital Ueno Clinic, 2-18-6, Higashi-Ueno, Taito-Ku, Tokyo 100-0015, Japan; cl-rece@aisei-byouin.or.jp; 3 Department of Clinical Health Science, Tokai University School of Medicine Tokai University Tokyo Hospital, 1-2-5 Yoyogi, Shibuya-ku, Tokyo 153-0065, Japan; chizumiy@tokai.ac.jp (C.Y.); y-nishizaki@tokai.ac.jp (Y.N.)

**Keywords:** staple food, glycemic index, insulin, legumes, functional food, sensory, breaking stress, breaking strain

## Abstract

Legumes are low-carbohydrate food and are abundant in dietary fiber. In order to provide a functional staple food that does not cause a rapid increase in postprandial blood glucose levels, four kinds of legumes were focused on as ingredients. Noodles made from dehulled yellow pea, unshelled yellow pea, chickpea, and lentil were prepared and evaluated as functional staple foods for controlling blood glucose via an in vitro digestion method. We also measured breaking stress and breaking strain using a creep meter, as well as sensory tests on a 9-point hedonic scale. The noodles made from yellow pea had high values for both breaking stress and breaking strain, and was highly regarded in the sensory tests. Therefore, the noodles made from yellow pea on postprandial glucose and insulin response were measured in a randomized double-blind study (*n* = 12). The results show that noodles made from yellow pea have a low glycemic index (50.4), and have potential as a functional staple food.

## 1. Introduction

A staple food is a food that people consume frequently, usually on a daily basis. Typical staple foods include grains (rice, wheat, etc.) and tubers (sweet potato, potato, cassava, etc.). Rice, when considered in combination with other crops, is the staple food of half the global population [1]. The next most common staple food is wheat, accounting for a relatively high percentage, followed by corn, sorghum, millet, and legumes [2].

Characteristically, the grains that play a central role as staple foods contain high levels of carbohydrates. Carbohydrates are digested and absorbed, which increases the blood’s glucose concentration. Blood glucose is an important source of energy for survival, but persistent excessive increases in blood glucose or chronic hyperglycemia, cause conditions, such as diabetes, coronary artery disease, and obesity, and are a global health risk. Past studies have shown that white rice, the world’s most widely consumed staple food, may increase the risk of type 2 diabetes when consumed in large amounts [3,4].

Glycemic Index (GI) is a metric describing the “postprandial increase in blood glucose”, which was proposed by Jenkins et al. [5] and is now an internationally standardized index. GI is defined as the ratio between the incremental area under the curve (IAUC) of the reference food and the IAUC after the intake of a test food containing a certain amount of carbohydrates up to 2 h after consumption. When glucose is set as the reference, a GI of 70 or higher is categorized as a high GI food, a GI of 56–69 is categorized as a medium GI food, and a GI of 55 or lower is categorized as a low GI food [6]. The FAO/WHO recommends a low GI diet to prevent diseases, such as diabetes, coronary artery disease, and obesity. It has also been reported in Japan that a low GI diet is beneficial for blood glucose control and improving dyslipidemia [7,8,9].

Legumes are known as typical low GI foods. The GI of white rice is approximately 80, but the GI of boiled chickpeas is 28 and the GI of other legumes is approximately 30 [10]. Legumes are high in carbohydrates but are also high in dietary fiber and resistant starch, which are not digested and absorbed, and are effective in suppressing postprandial glucose elevation [11]. Legumes are also rich in proteins, minerals, and phytochemicals, such as polyphenols, making them an excellent source of nutrients [12]. Furthermore, the proteins in peas and chickpeas have a lower risk of causing allergic diseases and a higher amino acid score in comparison to wheat gluten [13,14]. The active consumption of legumes is shown to have various beneficial effects, including maintaining satiety, reducing weight, preventing metabolic syndrome, and extending healthy life expectancy [15].

In this study, we prepared noodles exclusively from legumes as ingredients, to provide a functional staple food that is palatable and does not cause a rapid postprandial glucose increase. We conducted in vitro digestion experiments, sensory tests, and break tests to determine whether there were differences in legume varieties and compositions, and compared them with commercially available gluten-free pasta based on legumes.

## 2. Materials and Methods

### 2.1. Test Material

Dehulled yellow pea (Ohtsuya Inc., Tokyo, Japan), unshelled pea (Toyota Tsusho Foods Corp., Tokyo, Japan), chickpea (Bob’s Red Mill Inc., Milwaukee, OR, USA), and lentil (Beans Market Inc., Shizuoka, Japan) sold in Japan for food use were purchased. We then used these legume flours to prepare noodles using extrusion molds [16]. Briefly, 50% water was added to the legume flour, kneaded, extruded at 120 °C, and then air dried. In this study, two commercially available gluten-free pastas (CGF-A; Whole Foods Market., Austin, TX, USA, CGF-B; Explore Cuisine Inc., Red Bank, NJ, USA) containing legumes as ingredients were purchased and tested. Commercially available pressurized steamed white rice (WR; Sato Foods Co., Ltd., Niigata, Japan.) was used as the control food for the in vitro digestion and the GI measurement.

To cook the noodle products, dehulled yellow pea noodles (YP), unshelled yellow pea noodles (YP-U), chickpea noodles (CP), lentil noodles (LT), and commercially available gluten-free pasta, containing legumes as ingredients (CGF-A; red lentil, CGF-B; chickpea), were boiled in water containing 0.7% NaCl (YP, 8 min; YP-U, 6 min; CP, 8 min; LT, 7 min; CGF-A, 7 min; CGF-B, 7 min). The boiling time was evaluated in advance and set as the time that provided the best texture (data not shown). The WR was boiled in a hot water until it was edible.

The nutritional components of the prepared noodles were analyzed at the Japan Food Research Laboratories (JFRL) which is one of the world’s largest, testing service providers. Moisture was measured by a dry weight method. Protein was calculated from the total nitrogen quantified by the combustion method. Fat was measured by the Soxhlet extraction method using diethyl ether. Total dietary fiber was analyzed by enzymatic gravimetric methods. Available carbohydrate was obtained by difference, by subtracting the sum of grams of water, protein, fat, ash (data not shown), and dietary fiber. The product labels were referenced for the nutritional components of CGF-A, CGF-B, and WR. Table 1 shows the main nutritional components of the prepared noodles and the CGF-A and CGF-B.

### 2.2. In Vitro Digestion Experiment

Rapidly available glucose (RAG), which correlates with postprandial glycemic response, was measured in accordance with previous studies [17]. Briefly, the cooked samples were homogenized and kept at 70 °C in a hot water bath. Approximately 3 g samples were collected and cooled down to 37 °C, then treated for 30 min in 10 mL pepsin–guar gum solution (Sigma-Aldrich Co. LLC., St. Louis, MO, USA. P-7000). Five milliliters of aqueous sodium acetate solution (0.5 M) was added to neutralize, alongside 5mL enzyme mix solution containing pancreatin (Sigma-Aldrich Co. LLC., St. Louis, MO, USA. P-7545), amyloglucosidase (Sigma-Aldrich Co. LLC., St. Louis, MO, USA. A7420), and invertase (Sigma-Aldrich Co. LLC., St. Louis, MO, USA. 14504) for 20 min. During this time, the solution was agitated and stirred in a constant temperature water bath kept at 35 °C. The enzyme solution was prepared as described [17] immediately before the experiment. Then, the released glucose was quantified as RAG using high performance liquid chromatography (HPLC). The HPLC system consisted of a SHIMADZU LC-20 AD (SHIMADZU CORPORATION., Kyoto, Japan.) to Shodex RI-210H refractive index detector (Showa Denko K. K., Tokyo, Japan). Chromatographic experiments were performed in the isocratic mode. Shodex HILICpak VG-50 4E column (Showa Denko K. K., Tokyo, Japan, 4.6 × 250 mm, 5 μm particle) was used. The mobile phase consisted of a mixture of acetonitrile and water solution (80:20, *v*/*v*). The flow rate was set to 0.8 mL/min and the oven temperature to 35 °C. The injection volume was 10 µL.

### 2.3. Sensory Evaluation

Nine-point hedonic sensory evaluations were performed with 30 healthy men and women in accordance with previous studies [18]. Briefly, the noodles were cooked in the same manner as described in the “2.1. Test Material” section. Next, approximately 20 g of the cooked noodle samples were provided to each subject. Tasting was completed within 5 min of boiling. An evaluation was performed with the following: extremely dislike = 1 point, dislike very much = 2 points, moderately dislike = 3 points, slightly dislike = 4 points, neither like nor dislike = 5 points, like slightly = 6 points, like moderately = 7 points, like very much = 8 points, like extremely = 9 points. This method complies with ISO 8589: 2007. In this study, the products were evaluated on appearance, taste, aroma, texture, hardness, stickiness, and overall acceptance.

### 2.4. Physical Property Tests

The stress–strain curve was determined using the same method as Ogawa et al. [19]. Briefly, the products were boiled as described above, and breaking stress and breaking strain were measured immediately using a YAMADEN creep meter (YAMADEN Co., Ltd., Tokyo, Japan), with samples deformed to a strain rate of 90% using a 20 N load cell with a No. 49 wedge-shaped plunger at 0.5 mm/sec. The strain was normalized by the diameter of pasta. Ten noodles were measured for each sample. The samples were always kept at or above 60 °C and the measurements were completed within 10 min of boiling.

### 2.5. GI/insulin Measurement Subjects

GI measurements were performed using the method specified by Sugiyama et al. [20]. The subjects were healthy men and women between the ages of 20 and 49 with a body mass index (BMI) of 30 kg/m^2^ or less. The selected subjects did not have impaired glucose tolerance within the past 12 months, did not regularly use medication or health-claimed foods that intended to improve glycometabolism, and did not excessively smoke (those who do not smoke more than 20 cigarettes per day) or consume alcohol (those who do not consume more than 60 g of alcohol per day). The subjects provided informed consent in advance, in accordance with the Declaration of Helsinki, and the test was performed with the written informed consent of the subject.

### 2.6. Study Design

**Figure 1:**
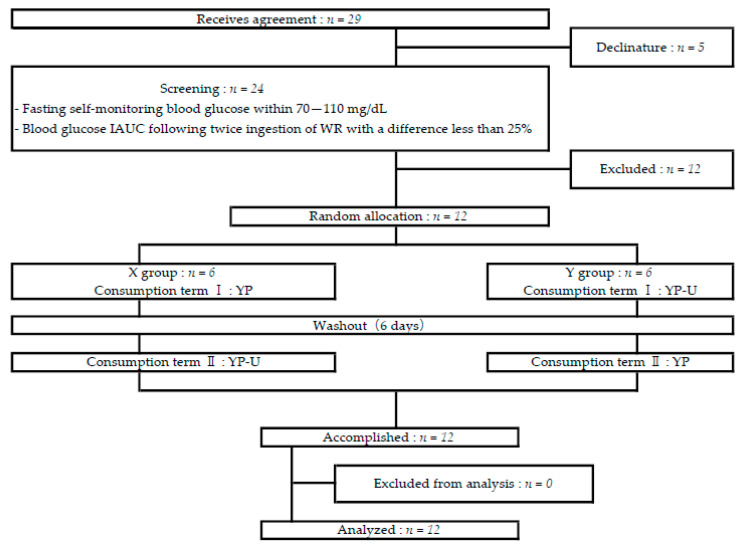
Flow chart showing subject classification. IAUC—Incremental Area Under the Curve, WR—white rice, YP—dehulled yellow pea noodles, YP-U—unshelled yellow pea noodles. X group consumed YP in term I and YP-U in Term II. Y group consumed YP-U in term I and YP in Term II.

GI and insulin measurements were performed using a randomized, double-blind, crossover design. Subjects who gave their consent were evaluated with a lifestyle questionnaire, interview, anthropometry, fasting blood sampling, and urine collection. The subjects were asked to finish their evening meal by 20:00 on the day before the preliminary test and the test food intake, and fasting was continued for at least 10 h before the test. The consumption of all food and drinks, including gum and candy, was restricted until all tests were completed on the day of the test, but water intake was permitted. Drinking alcohol on the day before and on the day of the test was prohibited. Fasting blood glucose levels were measured using a self-monitoring blood glucose meter (Accu-Chek Aviva; Roche DC Japan., Tokyo, Japan), and subjects within 70–110 mg/dL were selected. The selected subjects underwent two preliminary tests to determine the blood glucose IAUC following the ingestion of WR. If the difference between the IUAC of the preliminary tests exceeded 25%, a third preliminary examination was performed and subjects with a difference of less than 25% were selected. The interval(s) between the preliminary tests was at least one week in duration. Twelve people were enrolled in the study. The test food intake was divided into two sessions: intake period I and intake period II, with a washout period of six days set between the intakes. The study design and subjects are shown in Figure 1, and the nutritional composition of the test food is shown in Table 2.

The blood glucose levels used for calculating GI were measured using a self-monitoring blood glucose meter (Accu-Chek Aviva) before consumption, and 15, 30, 45, 60, 90, and 120 min after consumption. The IAUC was calculated. The GI calculation was performed using the following equation:

(1) GI value =(test food IAUC) ÷ (reference food IAUC)×100

The glucose based GI (GI_G_) was calculated in accordance with previous studies by multiplying the rice based GI (GI_R_), calculated based on the WR used as the reference food, by 0.8 [19]. Insulin was measured before consumption, 30 min, 60 min, and 120 min after consumption using chemiluminescence immunoassay (CLIA method) at the BML Inc.

This study was performed with the approval of the Ueno Clinic Institutional Review Board (IRB number: 12000071). The study protocol was pre-registered in the Clinical Trial Registry System (UMIN-CTR) (UMIN 000037071).

### 2.7. Statistical Analysis

In this study, data are provided as means ± SD. RAG, sensory evaluation scores, breaking stress and breaking strain, GI, blood glucose concentration, insulin concentration, blood glucose IAUC, and insulin IAUC were assessed using a one-way analysis of variance (ANOVA) with Bonferroni correction. The level of statistical significance was set at *p* ≤ 0.05 after Bonferroni correction. All calculations were performed by using the Bell Curve for Excel software (Social Survey Research Information Co., Ltd., Tokyo, Japan).

## 3. Results

### 3.1. In Vitro Digestion Experiment

Four types of noodles were prepared using legumes. We artificially digested these samples and measured the amount of RAG to estimate differences in postprandial blood glucose elevation. LT had the lowest RAG of the prepared pasta foods, at 7.53 ± 0.59%; CP had the highest, at 10.43 ± 1.22%. CGF-A and CGF-B had RAG levels of 9.91 ± 0.64% and 9.71 ± 0.58%, respectively, as shown in Table 3. Statistical analysis showed that RAG was significantly lower in all noodles than in WR. In a comparison of the noodles, there were no statistically significant differences.

### 3.2. Sensory Evaluation

A sensory evaluation of palatability was conducted to verify whether the four types of prepared noodles were superior or inferior in taste and whether they were better than existing commercial gluten-free pasta products. The results of the appearance, taste, aroma, texture, hardness, stickiness, as well as the overall acceptance, are shown in Table 4. The results of statistical analysis showed that the appearance of YP and CP was rated significantly higher than the other samples. In taste, YP and YP-U had significantly higher palatability than the existing commercial products, CGF-A and CGF-B. There was no significant difference in aroma among any of the products. YP, YP-U, CP, and LT had significantly better texture than the existing commercial products, CGF-A and CGF-B. YP, YP-U, and CP had significantly more palatable hardness than the existing commercial products, CGF-A and CGF-B. Stickiness and overall acceptance showed similar trends, with YP and CP having significantly higher palatability than LT, CGF-A, and CGF-B. In summary, YP, YP-U, and CP had higher palatability than CGF-A and CGF-B.

### 3.3. Physical Property Tests

**Figure 2:**
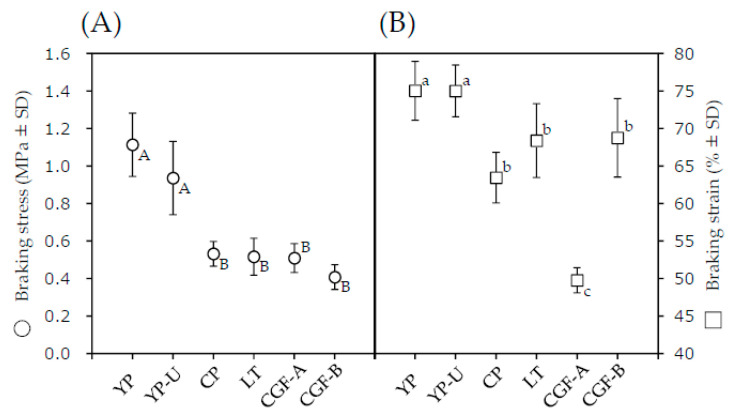
Breaking stress and breaking strain in legume-based noodles. (**A**) for breaking stress and (**B**) for breaking strain. All data are means ± standard deviation (SD) of 10 noodles. Uppercase letters are allocated to breaking stress, lowercase letters are allocated to breaking strain. Different letters were significantly different; *p* < 0.05 (Bonferroni correction).

The breaking stress and breaking strain of the prepared noodles were examined using a creep meter to quantitatively evaluate the texture, as shown in Figure 2. YP and YP-U had significantly higher breaking stress than the other samples, as shown in Figure 2A. This result indicates that YP and YP-U, unlike the other samples, require more force to chew. In addition, YP and YP-U had significantly higher breaking strain than other samples, as shown in Figure 2B. This result indicates that YP and YP-U need to be more deformed to bite off. In summary, YP and YP-U had a unique texture that was different to the other noodles.

### 3.4. GI/Insulin Measurements

Based on the above results, YP and YP-U were selected as candidates for functional staple foods, based on their palatability and mechanical properties. Accordingly, the GI and insulin responses of YP and YP-U were measured in eight men and four women. The background of the subjects enrolled in the study is shown in Table 5. All 12 subjects adhered to the testing procedures described above.

GI_R_ and GI_G_ are shown in Table 6. The GI_R_ of YP and YP-U were 50.4 and 68.8, respectively. Statistical analysis showed that the GI_R_ of YP and YP-U were significantly lower than WR, and there was no significant difference between the GI_R_ of YP and the GI_R_ of YP-U. The GI_G_ of YP and of YP-U were 40.3 and 55.0, respectively, indicating that they were both low GI foods. 

**Figure 3:**
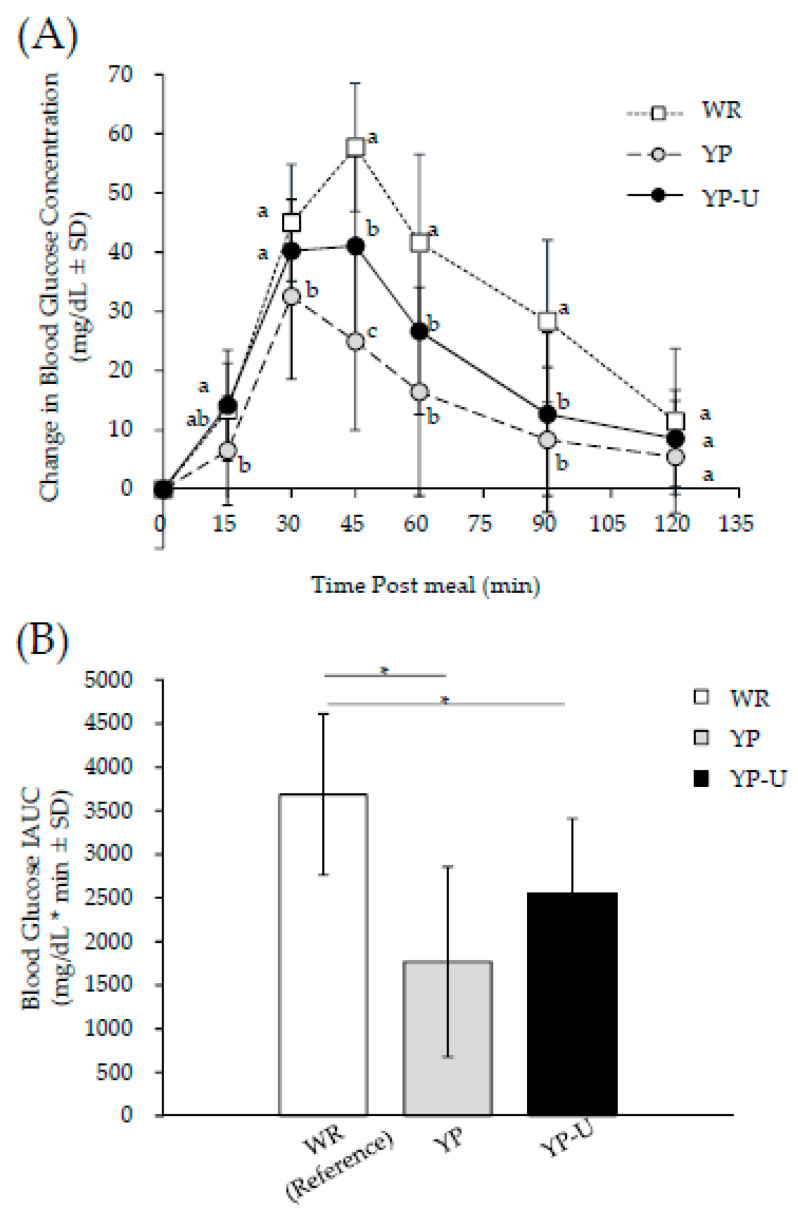
Changes of postprandial glycemic response and blood glucose IAUC. (**A**) For changes in postprandial blood glucose concentration. Different alphabets indicate significant differences; *p* < 0.05 (Bonferroni correction). (**B**) For blood glucose IAUC. * Significant difference between the comparison; *p* < 0.05 (Bonferroni correction). All data are means ± SD of 12 subjects.

The changes in blood glucose level at 15 min, 30 min, 45 min, 60 min, 90 min, and 120 min after consumption and the IAUC of blood glucose of WR, YP, and YP-U are shown in Figure 3. The change in blood glucose level at 15 min after consumption was significantly lower when YP was consumed compared to when YP-U was consumed. The change at 45 min, 60 min, and 90 min after loading was significantly lower after the consumption of both YP and YP-U compared to WR. The change in blood glucose level at 45 min was significantly lower after the consumption of YP compared to YP-U, as shown in Figure 3A. While the IAUC of blood glucose was significantly lower after the consumption of both YP and YP-U compared to consumption of WR, there were no statistically significant differences between IAUC after consumption of YP or YP-U, as shown in Figure 3B.

**Figure 4:**
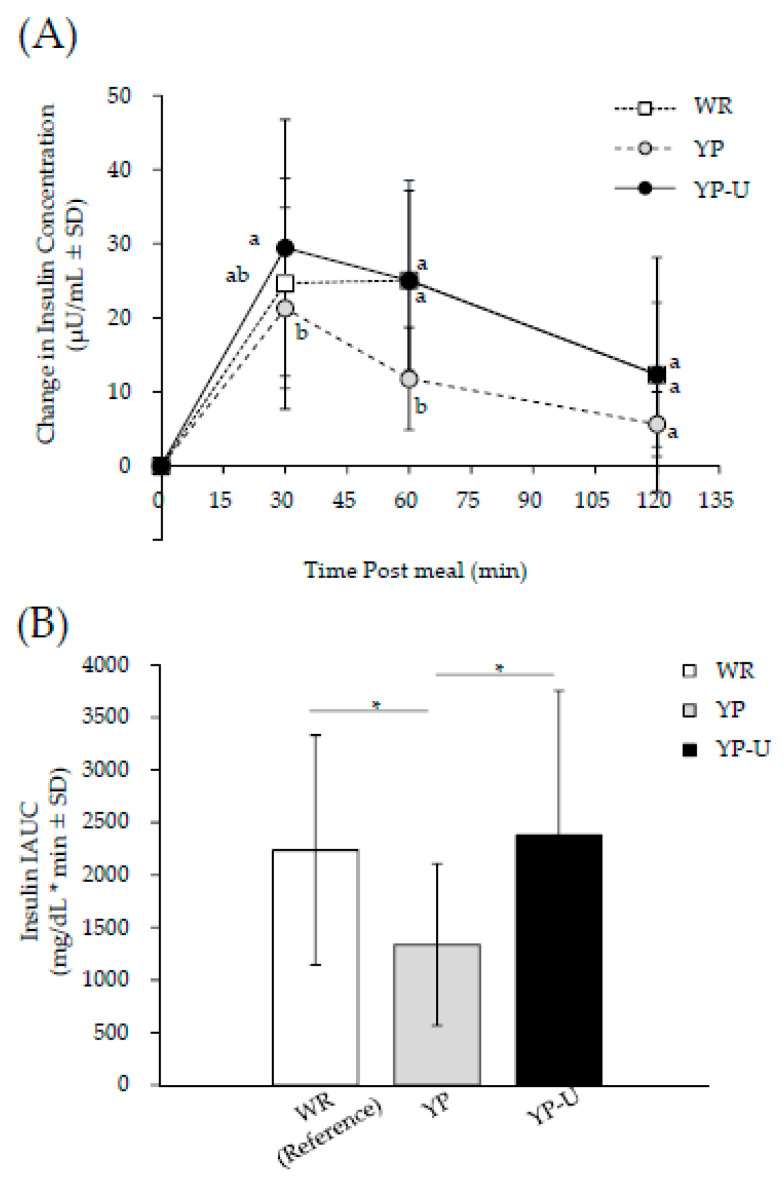
Changes of postprandial insulin response and insulin IAUC. (**A**) For changes in postprandial insulin concentration. Different alphabets indicate significant differences; *p* < 0.05 (Bonferroni correction). (**B**) For Insulin IAUC. * Significant difference between the comparison; *p* < 0.05 (Bonferroni correction). All data are means ± SD of 12 subjects.

The changes in insulin at 30 min, 60 min, and 120 min and the IAUC of insulin after the consumption of WR, YP, and YP-U are shown in Figure 4. Insulin levels at 30 min after loading were significantly higher when YP-U was consumed compared to the consumption of YP. At 60 min after consumption, the insulin levels were significantly lower when YP was consumed compared to the consumption of WR and YP-U, as shown in Figure 4A. The IAUC of insulin was significantly lower when YP was consumed, compared to the consumption of WR and YP-U, while there was no statistically significant difference in the IAUC of insulin between the consumption of YP-U and WR, as shown in Figure 4B.

## 4. Discussion

### 4.1. Rapidly Available Glucose, Sensory Evaluation, and Physical Properties of Noodles Made from Legumes

The purpose of this study was to provide a functional staple food that is palatable and does not cause a rapid postprandial glucose increase. Four types of noodles were prepared exclusively from legumes. First, an in vitro digestion experiment was performed to predict whether the variety and composition of the legumes used could cause a difference in postprandial blood glucose elevation. At the same time, the prepared noodles were compared with existing commercial gluten-free pasta products and WR.

The results of the in vitro digestion experiment showed no significant difference in RAG level between the legume noodles. RAG is correlated with postprandial blood glucose elevation. RAG is affected by the amount of carbohydrates and the structure of the food. For example, retrograded starch is less susceptible to hydrolysis by amylase, and only a small amount is available as RAG. Similarly, starch granules contained in legumes (mainly resistant starch; RS1 and RS2) are resistant to digestive enzymes [11,21,22]. The RAG values of peas, chickpeas, and lentils as unprocessed cooked beans are reported to be 6, 6, and 8 g/100g as eaten, respectively [17,23], which is consistent with the values of YP, YP-U, CP, and LT produced in this study. Furthermore, the carbohydrate content that affects RAG levels was similar among the various samples. This suggests that yellow peas, chickpeas, and lentils can be used to produce foods with similar postprandial blood glucose-elevating properties, regardless of the type of legume, and even if processed into extruded noodles.

To establish legume-based noodles as a staple food, its taste is important. It should be noted that all the noodles produced in this study had better palatability scores than products already on the market. Among the foods, YP, YP-U, and CP had relatively high palatability scores, suggesting that these noodles have the potential to be eaten daily. Furthermore, the overall palatability score of YP was 5.30 ± 0.24; however, it should be noted that five points corresponded to the evaluation of “Neither like nor dislike.” Crucially, if the palatability is too high, it may lead to overeating, while if it is too low, it may be difficult to continue eating. Therefore, we believe a score of this level is preferable for a staple food.

Some subjects commented in the sensory test that YP and YP-U had a unique al dente texture. Therefore, the mechanical properties were examined using a creep meter to quantitatively evaluate the texture. As a result, both breaking stress and breaking strain were found to be significantly larger in YP and YP-U than in other samples. This indicated that chewing noodles made with yellow peas requires more stress and deformation than chewing the other types. On the other hand, in the palatability sensory test, YP and YP-U were not the only foods that scored highly for “hardness” and “taste”, and there was no significant difference between the associated scores for YP, YP-U, and CP. This discrepancy is assumed to be a result of the multiple factors (other than the stress/strain characteristics) that affect palatability assessments.

### 4.2. Blood Glucose/Insulin Response from Noodles Made from Legumes

Based on these results, YP and YP-U were selected as study foods for measurements of GI and the post-consumption insulin responses in this study. In previous studies, the GI of noodles made with wheat and pea (70% and 30%, respectively) or wheat and lentil (50% each) were reported as 93 and 55, respectively, with a reference food of white bread [24,25]. These reports attempted to verify that the GI was reduced by mixing the main ingredient with legume; however, the GI was not significantly lower than the wheat-only control food [24,25]. Others have described noodles made exclusively from legumes, but these reports did not define the quality of taste or GI [26]. In order to increase the knowledge of legume-based products, this study evaluated the sensory, measured GI, and assessed post-consumption insulin response. The noodles made exclusively from yellow pea were shown to be a low GI food, indicating that it could be a staple food option for controlling blood glucose.

Although there was no significant difference in glycemic response between subjects when comparing YP and YP-U, YP was more effective in suppressing the increase in blood glucose. We hypothesized that YP-U would be more effective at suppressing the increase in blood glucose because it contains the seed coat of the legume, and therefore contains more dietary fiber [27], but no such effect was observed. This may be due to a weakening of the structural strength of the coat when processing into noodles, which made it easier to be digested in the stomach and intestine. The pea seed coat has a cuticle layer with a waxy surface [28]. It is presumed that the wax reduced the binding strength to the starch and proteins that form the structure of the noodles. If YP-U is easier to be digested, it is assumed that more glucose may be taken into the blood compared to YP. On the other hand, the equivalent RAG levels of YP and YP-U may indicate that the structural strength of the noodles was not relevant to the results, since homogenized samples were used for the in vitro digestion experiment. There was no difference between YP and YP-U in the measurement of mechanical properties, and there was no difference in the scores for the sensory test of texture, so the change in structural strength caused by the seed coat is assumed to be present primarily at a micro level (and undetectable through macro-structural determinations). If strong-bond noodles could be made through future improvements in manufacturing, it could be possible to provide superior foods for controlling blood glucose.

Blood glucose levels are affected by insulin secretion. In this study, blood insulin levels were measured at the same time as blood glucose. Insulin hypersecretion was significantly suppressed by YP compared with WR and YP-U. YP suppressed both blood glucose elevation and insulin hypersecretion, suggesting that it has excellent characteristics for blood glucose control. On the other hand, YP-U resulted in insulin secretion equal to that of WR. A GI study of bread made with chickpea reported that the addition of legumes increased insulin secretion [29]. Proteins [30] and the amino acids arginine [31] and phenylalanine [32] have been reported as food components, other than blood glucose, that increase insulin secretion. Peas are generally reported to contain a large amount of arginine and phenylalanine [33]. In this study, the YP-U group contained 2 g more protein than the YP group, which may have affected insulin secretion. YP-U produced the same level of insulin secretion as WR, but the blood glucose elevation was significantly lower than WR. Therefore, although YP-U is not as effective as YP, it is an advantageous food for controlling blood glucose. Further research on foods using pea products for glycemic control is necessary to fully understand their impact and relationship to human health.

In this study, the superiority of noodles made with peas in controlling blood glucose was discussed on the basis of GI, but glycemic load (GL) must also be considered. Many studies have simultaneously examined the relationship between GL and lifestyle-related disease factors in addition to GI [6]. GI is a “quality” index that indicates how easily sugar is absorbed, whereas GL is calculated by multiplying the amount of carbohydrates actually consumed in one meal by the GI of the food. Therefore, GL is an index that simultaneously represents “quality and quantity” [34]. In this study, subjects consumed 50 g of carbohydrates to measure GI, but to achieve this level of consumption, an intake of 216 g in wet weight was required for YP and 237 g for YP-U. This amount was approximately 1.5 times greater than the 147 g required for WR. As the difference between the calorific value per weight of YP, YP-U, and WR is small (WR = 624 kJ/100g, YP = 637 kJ/100g, YP-U = 605 kJ/100g), the consumption of these foods may have a more pronounced effect on glycemic control if the intake of YP and YP-U per meal was the same as that for WR.

This study does, however, have a few limitations. First, the sensory tests of noodles made with legumes were performed only by Japanese panelists; therefore, it is undeniable that there is a bias towards Japanese eating habits and preferences. Second, GI and insulin secretion measurements using YP and YP-U were also performed with Japanese patients. Thus, the response to YP and YP-U intake may not account for racial differences in consumers. Future studies on the palatability, the blood glucose levels, and the insulin levels associated with noodles made with yellow peas should be conducted with non-Japanese subjects. Additionally, further research is needed to incorporate these foods into the diet in various parts of the world. Third, we measured GI and insulin secretion in healthy men and women, but we have not been able to verify whether these foods are effective for diabetic or pre-diabetic patients who wish to control blood glucose. Therefore, it is desirable to verify the effect of these foods on glycemic control in non-healthy subjects. Fourth, as the study adopted a single-dose study design, it is not clear how long-term continuous intake would affect glycemic control. Therefore, long-term intervention tests are needed. Finally, in this study, different types of legumes were used to produce noodles, but it may not be possible to optimize the production method. The GI and insulin response may change if the manufacturing conditions change, so further research is needed to evaluate the influence of production method on the development of superior foods.

## 5. Conclusions

In this study, noodles were prepared from legumes in order to provide a novel functional staple food. In vitro digestion, breaking stress and strain measurement, and sensory testing were performed. Our results indicate that using yellow pea as the exclusively ingredient could provide palatable noodles with a unique texture from other legume-based noodles. Subsequently, GI and postprandial insulin responses were measured, which indicated that YP and YP-U have low GI and insulin saving properties. Therefore, we conclude that noodles exclusively made from yellow peas have the potential to be a novel staple food with the glycemic control function. If further improvements in taste and function are achieved, then noodles exclusively made from yellow pea might become a substitute for rice and wheat, and could help reduce diseases caused by high blood glucose.

## Figures and Tables

**Table 1:** Nutritional composition of legume-based noodles (grams per 100 g, as eaten).

Sample	Moisture	Protein	Fat	Carbohydrate	Total Dietary Fiber
YP	59.6	10.1	1.0	23.1	5.3
YP-U	60.7	8.7	1.0	20.8	7.8
CP	63.5	8.0	2.1	20.8	4.6
LT	64.4	9.3	0.6	17.6	6.8
CGF-A	61.9	10.7 ^†^	0.7 ^†^	21.6 ^†^	4.0 ^†^
CGF-B	64.6	7.9 ^†^	1.8 ^†^	21.2 ^†^	3.6 ^†^

^†^ Referenced from product nutrition facts. Unmarked figures were measured at Japan Food Research Laboratories. YP—dehulled yellow pea noodles, YP-U—unshelled yellow pea noodles, CP—chickpea noodles, LT—lentil noodles, CGF-A—commercially available gluten-free pasta A, CGF-B—commercially available gluten-free pasta B.

**Table 2:** Nutritional composition of glycemic index test foods.

Nutritional Composition	WR (Reference)	YP	YP-U
Energy (kJ) ^a^	917	1394	1425
Protein (g)	3 ^†^	22	21
Fat (g)	0 ^†^	2	2
Total dietary fiber (g)	2	12	18
Available carbohydrate (g)	50 ^†^	50	50

^†^ Referenced from product nutrition facts. ^a^ Calculated as protein = 17 kJ/g, fat = 37 kJ/g, available carbohydrate = 17 kJ/g, and total dietary fiber = 8 kJ/g. Unmarked figures were measured at Japan Food Research Laboratories.

**Table 3:** Rapidly available glucose content in legume-based noodles and white rice (grams per 100 g, as eaten).

Sample	RAG (%)
YP	8.34 ± 1.07
YP-U	8.20 ± 0.88
CP	10.43 ± 2.99
LT	7.53 ± 1.46
CGF-A	9.91 ± 1.56
CGF-B	9.71 ± 1.42
WR	17.32 ± 3.69 *

All the values are means ± standard deviation (SD) of six determinations. RAG—rapidly available glucose, WR—white rice. * Significant difference between WR and YP, YP-U, CP, LT, CGF-A, and CGF-B; *p* < 0.05 (Bonferroni correction).

**Table 4:** Sensory evaluation scores for legume-based noodles.

Sample	Appearance	Taste	Aroma	Texture	Hardness	Stickiness	Overall Acceptance
YP	6.23 ± 1.07 ^a^	5.60 ± 1.25 ^a^	4.70 ± 1.39 ^a^	5.20 ± 1.63 ^a^	5.23 ± 1.65 ^a^	5.67 ± 1.40 ^a^	5.30 ±1.32 ^a^
YP-U	3.90 ± 1.32 ^b^	4.80 ± 1.03 ^a^	4.10 ± 1.24 ^a^	4.87 ± 1.33 ^a^	5.40 ± 1.28 ^a^	5.23 ± 1.22 ^ab^	4.57 ± 1.28 ^ab^
CP	5.23 ± 1.63 ^a^	4.77 ± 1.76 ^ab^	4.60 ± 1.29 ^a^	5.60 ± 2.03 ^a^	5.50 ± 1.96 ^a^	5.60 ± 1.81 ^a^	4.97 ± 1.94 ^a^
LT	3.20 ± 1.61 ^b^	4.60 ± 1.28 ^ab^	4.63 ± 1.60 ^a^	4.80 ± 1.45 ^ac^	4.73 ± 1.51 ^ab^	4.37 ± 1.50 ^b^	3.80 ± 1.40 ^b^
CGF-A	3.80 ± 1.37 ^b^	4.10 ± 1.56 ^b^	4.10 ± 1.36 ^a^	4.73 ± 1.51 ^b^	3.17 ± 1.57 ^b^	3.57 ± 1.23 ^c^	2.93 ± 1.32 ^c^
CGF-B	4.00 ± 1.11 ^b^	3.73 ± 1.80 ^b^	3.93 ± 1.56 ^a^	3.60 ± 1.61 ^bc^	4.10 ± 1.47 ^b^	4.00 ± 1.72 ^c^	3.40 ± 1.67 ^c^

All the values are means ± standard deviation SD of thirty individual determinations. In each evaluation, values with different alphabets were significantly different; *p* < 0.05 (Bonferroni correction).

**Table 5:** Descriptive characteristics of study subjects.

Characteristic	Mean ± SD	Range of Values
Age (yrs)	37.8 ± 9.5	21–47
Weight (kg)	66.9 ± 12.6	47.9–91.9
Height (cm)	170.5 ± 9.9	156.7–185.6
BMI (kg/m^2^)	22.9 ± 3.5	18.3–28.7
Systolic blood pressure (mmHg)	121.1 ± 12.8	100–145
Diastolic blood pressure (mmHg)	73.3 ± 13.5	52–105
Fasting blood glucose (mg/dL)	92.3 ± 5.2	86–104
Triglyceride (mg/dL)	81.7 ± 35.8	27–133
Total cholesterol (mg/dL)	178.4 ± 17.0	146–199
HDL-C (mg/dL)	62.6 ± 17.9	42–99
LDL-C (mg/dL)	97.9 ± 20.1	73–143

All the values are means ± SD of 12 subjects. BMI—Body mass index, HDL-C—High density lipoprotein cholesterol, LDL-C—Low density lipoprotein cholesterol.

**Table 6:** Glycemic index of yellow pea-based noodles.

Sample	GI_R_	GI_G_^#^
WR (Reference)	100 *	80
YP	50.4 ± 31.6	40.3 ± 25.3
YP-U	68.8 ± 12.4	55.0 ± 9.92

All the values are means ± SD of 12 subjects. GI_R_—rice based glycemic index, GI_G_—glucose based glycemic index. ^#^ Values converted from GI_R_ by multiplying by a factor of 0.8. * Significantly difference between WR and YP and YP-U; *p* < 0.05 (Bonferroni correction).
